# μ-1,6-Dioxo-1,6-di­phenyl­hexane-3,4-diolato-bis­[(2,2′-bi­pyridine)­chlorido­copper(II)] dihydrate

**DOI:** 10.1107/S2414314623007137

**Published:** 2023-08-30

**Authors:** Luke Nye, Shane G. Telfer, Mark M. Turnbull

**Affiliations:** a Carlson School of Chemistry and Biochemistry, Clark University, 950 Main St., Worcester, MA 01610, USA; b School of Natural Sciences, Institute of Fundamental Sciences, Massey University, Palmerston North, New Zealand; Sunway University, Malaysia

**Keywords:** Cu^II^, hydrate, hydrogen-bonding, tetronedioate, crystal structure

## Abstract

The reaction of CuCl_2_ with 1,6-diphenyl-1,3,5,6-hexa­netetrone and 2,2′-bi­pyridine (bipy) in ethanol gave crystals of the corresponding bimetallic complex, [Cu_2_(C_18_H_12_O_4_)Cl_2_(C_10_H_8_N_2_)_2_]·2H_2_O. The mol­ecule is centrosymmetric with each Cu^II^ ion coordinated to two oxygen atoms from the tetronediate, two nitro­gen atoms from a bipy ligand and one coordinated chloride ion. A water mol­ecule of crystallization forms hydrogen bonds to the chloride ions, linking the mol­ecules into a chain parallel to the *bc*-face diagonal.

## Abstract 2

The title complex sits astride an inversion center with each Cu^II^ atom having an N_2_O_2_Cl coordination sphere. Charge balance is achieved *via* the tetronediate and one coordinated chloride ion per Cu^II^. Hydrogen bonding to a solvent water mol­ecule links the mol­ecules into a chain parallel to the *bc*-face diagonal.

## Graphical abstract

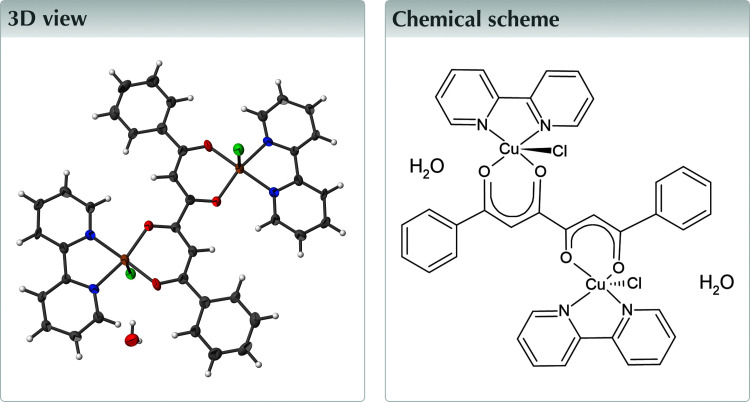

## Structure description

1,6-Diphenyl-1,3,4,6-hexa­netetrone has been known for over 100 years (Widman & Virgin, 1909 ▸) and its structure has been reported (Kaitner *et al.*, 1992 ▸). Both its synthesis by oxidation (Balenović *et al.*, 1954 ▸) and its reactions with oxidizing agents have been studied (Balenović, 1948 ▸; Bird & Thorley, 1977 ▸; Poje *et al.*, 1978 ▸), as well as its use as a starting material for the preparation of a variety of aminated products (Lacan *et al.*, 1973 ▸; Unterhalt & Pindur, 1977 ▸; Kaitner *et al.*, 1992 ▸; Waring *et al.*, 2002 ▸; Kobelev *et al.*, 2019 ▸). The backbone core resembles a bis-acac structure and and as such it has been used in the preparation of transition-metal complexes (Boucher & Bailar, 1964 ▸; Saalfrank *et al.*, 1998 ▸; Nawar, 1994 ▸). However, we were surprised to find that there are no reported structures of transition-metal complexes containing this ligand (Groom *et al.*, 2016 ▸). We investigated its coordination chemistry with Cu^II^ as part of our studies on potential magnetic ladders (Monroe *et al.*, 2022 ▸).

**Figure 1:**
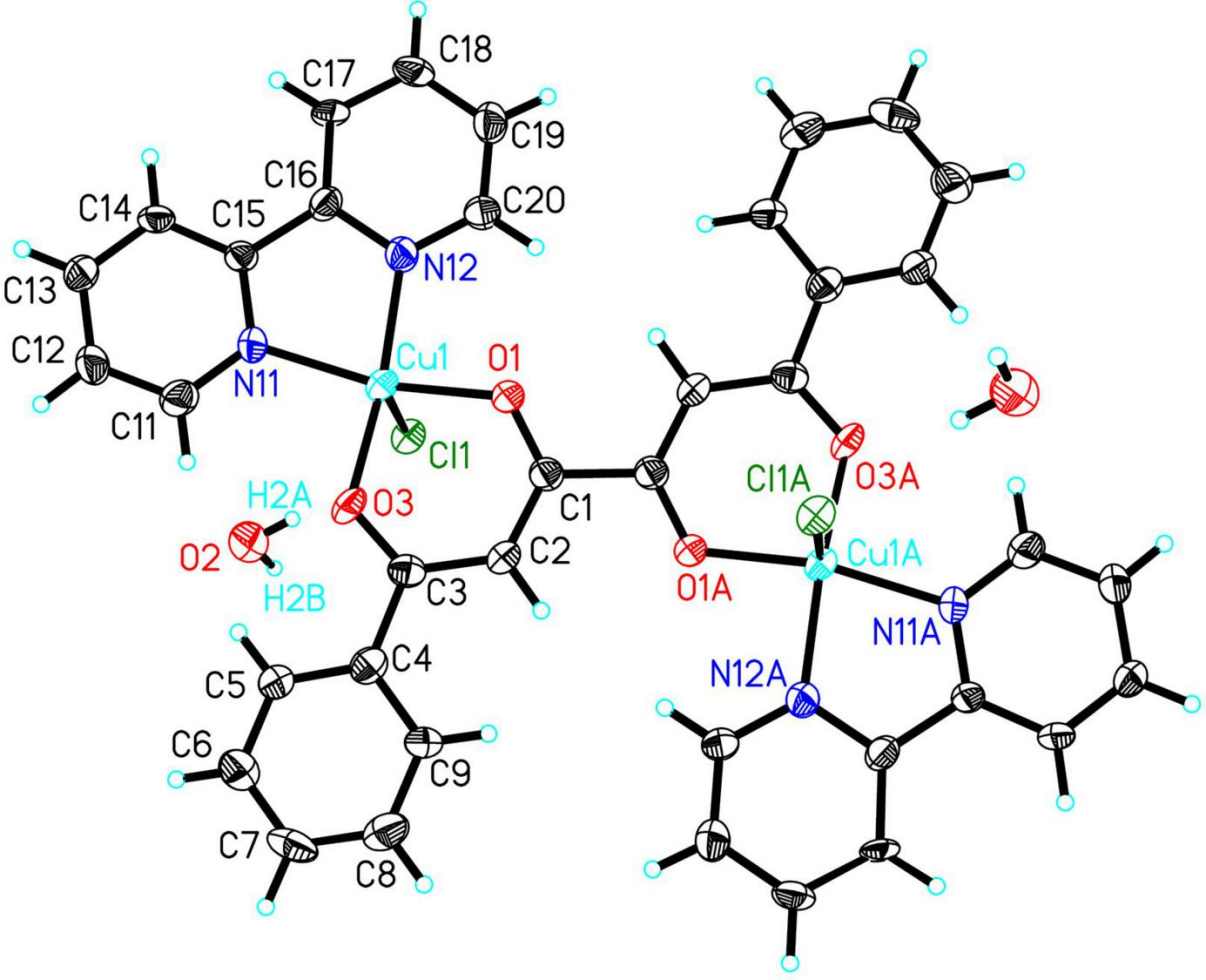
The mol­ecular structure of the title compound. Only the asymmetric unit and Cu coordination spheres are labeled. Hydrogen atoms are shown as spheres of arbitrary size. Symmetry operation B: −*x*, 1 − *y*, 2 − *z*.

The mol­ecule sits astride a crystallographic inversion center (Fig. 1 ▸). Each Cu^II^ ion is five-coordinate, including two oxygen atoms from the tetronediate ligand, two nitro­gen atoms from a bipy mol­ecule and one coordinated chloride ion. The CuN_2_O_2_ equatorial plane is nearly planar (mean deviation of the N_2_O_2_ donor set = 0.0219 Å) with the Cu^II^ ion displaced [0.2219 (15) Å] toward the chloride ion. The 1,3-dionato motif chelates a copper ion and generates a six-membered metallocyclic ring that is only slightly less planar (mean deviation of constituent atoms = 0.1207 Å). The two heterocyclic rings are co-planar as required by symmetry. The pyridyl rings are canted 0.67 (13)° from each other.

**Figure 2:**
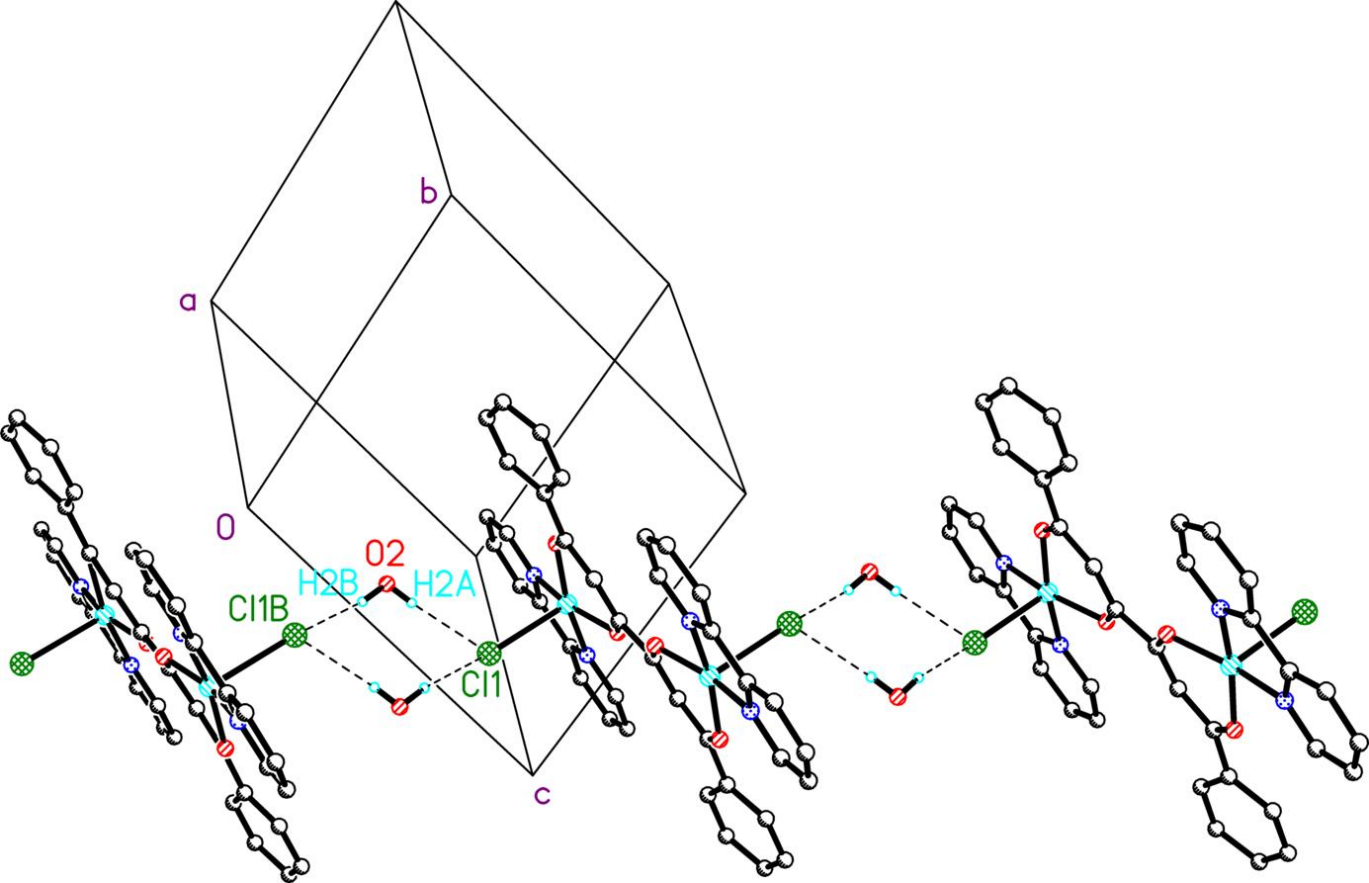
Chain formation *via* hydrogen bonding. Symmetry operation B: −*x*, −*y*, 1 − *z*.

π-Stacking is observed between mol­ecules. The Cu1-dionato ring sits above the N11-containing bpy ring with an inter­planar distance of 3.33 (2) Å; the rings are canted 4.4 (2)° with respect to each other. The distance between the ring centroids is 3.73 (2) Å with a slip angle of 25.1 (3)°. This effectively blocks the vacant coordination site on Cu1, preventing the addition of a sixth ligand. π-Stacking is also observed between the phenyl rings of the tetrone ligand. Adjacent phenyl rings are parallel with an inter­planar distance of 3.33 (2) Å with a slip angle of 22.8 (3)°. The bimetallic units are linked into chains *via* hydrogen bonds between the solvent water mol­ecules and chloride ions (Table 1 ▸ and Fig. 2 ▸).

## Synthesis and crystallization

CuCl_2_ (0.403 g, 2.99 mmol) was dissolved in 50 ml of absolute ethanol to generate a green solution. 2,2′-Bi­pyridine (0.469 g, 3.01 mmol) was added to the solution with stirring to make a light-blue slurry. Addition of 1,6-diphenyl-1,3,4,6-hexa­ne­tetrone (0.438 g, 1.49 mmol) generated a lime green slurry, which was stirred for 1 h. The precipitate was recovered by vacuum filtration, washed with ethanol and dried in air to yield 0.973 g of lime green powder (77%). Crystals suitable for X-ray diffraction were grown by recrystallization from DMF.

## Refinement

Crystal data, data collection and structure refinement details are summarized in Table 2 ▸. Eight reflections were omitted from the final refinement owing to poor agreement; details are included in the CIF.

## Supplementary Material

Crystal structure: contains datablock(s) I. DOI: 10.1107/S2414314623007137/tk4095sup1.cif

Structure factors: contains datablock(s) I. DOI: 10.1107/S2414314623007137/tk4095Isup2.hkl

CCDC reference: 2288356

Additional supporting information:  crystallographic information; 3D view; checkCIF report

## Figures and Tables

**Table 1:** Hydrogen-bond geometry (Å, °)

*D*—H⋯*A*	*D*—H	H⋯*A*	*D*⋯*A*	*D*—H⋯*A*
O2—H2*A*⋯Cl1	0.82 (5)	2.41 (5)	3.224 (3)	171 (5)
O2—H2*B*⋯Cl1^i^	0.98 (4)	2.33 (5)	3.307 (4)	172 (4)

Symmetry code: (i) 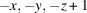 .

**Table 2:** Experimental details

Crystal data	
Chemical formula	[Cu_2_(C_18_H_12_O_4_)Cl_2_(C_10_H_8_N_2_)_2_]·2H_2_O
*M*_r_	838.65
Crystal system, space group	Triclinic, *P* 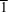
Temperature (K)	123
*a*, *b*, *c* (Å)	8.7211 (2), 10.4194 (2), 10.5243 (7)
α, β, γ (°)	106.426 (7), 109.090 (8), 90.373 (6)
*V* (Å^3^)	861.67 (8)
*Z*	1
Radiation type	Cu *K*α
μ (mm^−1^)	3.41
Crystal size (mm)	0.13 × 0.09 × 0.02

Data collection	
Diffractometer	Rigaku Spider
Absorption correction	Multi-scan (*ABSCOR*; Rigaku, 1995 ▸)
*T*_min_, *T*_max_	0.641, 0.929
No. of measured, independent and observed [*I* > 2σ(*I*)] reflections	7470, 2552, 1901
*R*_int_	0.046
θ_max_ (°)	61.1
(sin θ/λ)_max_ (Å^−1^)	0.568

Refinement	
*R*[*F* ^2^ > 2σ(*F* ^2^)], *wR*(*F* ^2^), *S*	0.047, 0.121, 1.13
No. of reflections	2552
No. of parameters	241
H-atom treatment	H atoms treated by a mixture of independent and constrained refinement
Δρ_max_, Δρ_min_ (e Å^−3^)	0.57, −0.49

Computer programs: *CrystalClear* (Rigaku, 2005 ▸), *PROCESS-AUTO* (Rigaku, 1998 ▸), *SHELXS97* (Sheldrick, 2008 ▸), and *SHELXL2018/3* (Sheldrick, 2015 ▸), *XP* (Sheldrick, 2008 ▸).

## full crystallographic data

### Crystal data

**Table:**

[Cu_2_(C_18_H_12_O_4_)Cl_2_(C_10_H_8_N_2_)_2_]·2H_2_O	*Z* = 1
*M**_r_* = 838.65	*F*(000) = 428
Triclinic, *P*1	*D*_x_ = 1.616 Mg m^−^^3^
*a* = 8.7211 (2) Å	Cu *K*α radiation, λ = 1.54178 Å
*b* = 10.4194 (2) Å	Cell parameters from 3450 reflections
*c* = 10.5243 (7) Å	θ = 6.6–72.0°
α = 106.426 (7)°	µ = 3.41 mm^−^^1^
β = 109.090 (8)°	*T* = 123 K
γ = 90.373 (6)°	Plate, green
*V* = 861.67 (8) Å^3^	0.13 × 0.09 × 0.02 mm

### Data collection

**Table:**

Rigaku Spider diffractometer	2552 independent reflections
Radiation source: rotating anode	1901 reflections with *I* > 2σ(*I*)
Confocal optics monochromator	*R*_int_ = 0.046
Detector resolution: 10 pixels mm^-1^	θ_max_ = 61.1°, θ_min_ = 6.6°
ω–scans	*h* = −9→9
Absorption correction: multi-scan (ABSCOR; Rigaku, 1995)	*k* = −8→11
*T*_min_ = 0.641, *T*_max_ = 0.929	*l* = −11→11
7470 measured reflections	

### Refinement

**Table:**

Refinement on *F*^2^	Primary atom site location: structure-invariant direct methods
Least-squares matrix: full	Secondary atom site location: difference Fourier map
*R*[*F*^2^ > 2σ(*F*^2^)] = 0.047	Hydrogen site location: mixed
*wR*(*F*^2^) = 0.121	H atoms treated by a mixture of independent and constrained refinement
*S* = 1.13	*w* = 1/[σ^2^(*F*_o_^2^) + (0.0514*P*)^2^ + 0.1469*P*] where *P* = (*F*_o_^2^ + 2*F*_c_^2^)/3
2552 reflections	(Δ/σ)_max_ < 0.001
241 parameters	Δρ_max_ = 0.57 e Å^−^^3^
0 restraints	Δρ_min_ = −0.49 e Å^−^^3^

### Special details

**Table:**

Geometry. All esds (except the esd in the dihedral angle between two l.s. planes) are estimated using the full covariance matrix. The cell esds are taken into account individually in the estimation of esds in distances, angles and torsion angles; correlations between esds in cell parameters are only used when they are defined by crystal symmetry. An approximate (isotropic) treatment of cell esds is used for estimating esds involving l.s. planes.
Refinement. Hydrogen atoms bonded to carbon atoms were placed geometrically and refined with fixed isotropic thermal parameters. Hydrogen atoms bonded to O2 were located in the difference map and their positions refined with fixed isotropic thermal parameters (O—H = 0.82 (5) & 0.98 (4) Å).

### Fractional atomic coordinates and isotropic or equivalent isotropic displacement parameters (Å^2^)

**Table:**

	*x*	*y*	*z*	*U*_iso_*/*U*_eq_	
Cu1	0.33000 (7)	0.32164 (6)	0.96525 (6)	0.0307 (2)	
Cl1	0.11639 (12)	0.13154 (10)	0.77171 (10)	0.0345 (3)	
O1	0.1754 (3)	0.4177 (3)	1.0408 (3)	0.0324 (7)	
C1	0.0704 (5)	0.4796 (4)	0.9723 (4)	0.0254 (10)	
C2	0.0728 (5)	0.5143 (4)	0.8569 (4)	0.0294 (10)	
H2	−0.017613	0.555390	0.812000	0.035*	
O3	0.3211 (3)	0.4301 (3)	0.8421 (3)	0.0330 (7)	
C3	0.2001 (5)	0.4937 (4)	0.7987 (4)	0.0271 (10)	
C4	0.1975 (5)	0.5506 (4)	0.6837 (4)	0.0303 (10)	
C5	0.2992 (5)	0.5074 (4)	0.6059 (4)	0.0329 (11)	
H5	0.372026	0.443341	0.628745	0.039*	
C6	0.2963 (5)	0.5555 (5)	0.4963 (5)	0.0411 (12)	
H6	0.365425	0.523306	0.443454	0.049*	
C7	0.1939 (6)	0.6503 (5)	0.4627 (5)	0.0422 (12)	
H7	0.193055	0.683511	0.387222	0.051*	
C8	0.0922 (5)	0.6969 (4)	0.5389 (5)	0.0393 (12)	
H8	0.022438	0.763064	0.516788	0.047*	
C9	0.0926 (5)	0.6469 (4)	0.6470 (4)	0.0310 (10)	
H9	0.020939	0.677797	0.697730	0.037*	
N11	0.5341 (4)	0.2451 (3)	0.9445 (3)	0.0282 (8)	
C11	0.6014 (5)	0.2564 (4)	0.8501 (4)	0.0339 (11)	
H11	0.549680	0.303493	0.785150	0.041*	
C12	0.7425 (5)	0.2020 (4)	0.8441 (4)	0.0343 (11)	
H12	0.788780	0.212980	0.777127	0.041*	
C13	0.8170 (5)	0.1309 (4)	0.9368 (4)	0.0302 (10)	
H13	0.914522	0.092053	0.933922	0.036*	
C14	0.7475 (5)	0.1177 (4)	1.0326 (4)	0.0277 (10)	
H14	0.796296	0.069566	1.097313	0.033*	
C15	0.6054 (5)	0.1754 (4)	1.0339 (4)	0.0237 (9)	
C16	0.5227 (5)	0.1699 (4)	1.1351 (4)	0.0275 (10)	
C17	0.5733 (5)	0.1040 (4)	1.2364 (4)	0.0273 (10)	
H17	0.667767	0.057482	1.244906	0.033*	
C18	0.4850 (5)	0.1061 (4)	1.3261 (4)	0.0318 (11)	
H18	0.519826	0.063659	1.398103	0.038*	
C19	0.3447 (5)	0.1721 (4)	1.3075 (4)	0.0362 (11)	
H19	0.280783	0.173765	1.365635	0.043*	
C20	0.2994 (5)	0.2349 (4)	1.2041 (4)	0.0316 (11)	
H20	0.203908	0.280215	1.193139	0.038*	
N12	0.3846 (4)	0.2349 (3)	1.1175 (3)	0.0282 (8)	
O2	0.2128 (4)	0.0355 (4)	0.4911 (4)	0.0538 (10)	
H2A	0.178 (6)	0.059 (5)	0.557 (5)	0.065*	
H2B	0.110 (6)	−0.013 (4)	0.420 (5)	0.065*	

### Atomic displacement parameters (Å^2^)

**Table:**

	*U*^11^	*U*^22^	*U*^33^	*U*^12^	*U*^13^	*U*^23^
Cu1	0.0320 (4)	0.0347 (4)	0.0347 (4)	0.0178 (3)	0.0164 (3)	0.0183 (3)
Cl1	0.0317 (6)	0.0400 (7)	0.0367 (6)	0.0129 (5)	0.0136 (5)	0.0164 (5)
O1	0.0366 (17)	0.0391 (18)	0.0347 (17)	0.0202 (14)	0.0200 (14)	0.0217 (14)
C1	0.030 (2)	0.022 (2)	0.027 (2)	0.0052 (19)	0.012 (2)	0.0094 (19)
C2	0.027 (2)	0.034 (3)	0.032 (2)	0.0130 (19)	0.011 (2)	0.016 (2)
O3	0.0297 (16)	0.0351 (17)	0.0388 (17)	0.0198 (14)	0.0135 (13)	0.0156 (14)
C3	0.031 (3)	0.024 (2)	0.026 (2)	0.0026 (19)	0.009 (2)	0.0098 (19)
C4	0.025 (2)	0.032 (3)	0.030 (2)	0.0030 (19)	0.0050 (19)	0.009 (2)
C5	0.029 (2)	0.039 (3)	0.038 (3)	0.011 (2)	0.014 (2)	0.021 (2)
C6	0.039 (3)	0.050 (3)	0.042 (3)	0.004 (2)	0.018 (2)	0.021 (2)
C7	0.052 (3)	0.046 (3)	0.036 (3)	−0.005 (2)	0.013 (2)	0.025 (2)
C8	0.036 (3)	0.035 (3)	0.048 (3)	0.008 (2)	0.008 (2)	0.020 (2)
C9	0.031 (2)	0.038 (3)	0.030 (2)	0.008 (2)	0.010 (2)	0.020 (2)
N11	0.027 (2)	0.032 (2)	0.031 (2)	0.0111 (16)	0.0163 (17)	0.0114 (17)
C11	0.038 (3)	0.032 (3)	0.034 (3)	0.013 (2)	0.014 (2)	0.012 (2)
C12	0.038 (3)	0.037 (3)	0.037 (3)	0.012 (2)	0.022 (2)	0.013 (2)
C13	0.032 (2)	0.028 (2)	0.036 (3)	0.0114 (19)	0.015 (2)	0.013 (2)
C14	0.034 (2)	0.028 (2)	0.027 (2)	0.0105 (19)	0.011 (2)	0.017 (2)
C15	0.028 (2)	0.021 (2)	0.025 (2)	0.0064 (18)	0.0106 (19)	0.0097 (19)
C16	0.024 (2)	0.027 (2)	0.034 (2)	0.0078 (18)	0.0102 (19)	0.013 (2)
C17	0.034 (2)	0.024 (2)	0.034 (3)	0.0095 (19)	0.011 (2)	0.023 (2)
C18	0.043 (3)	0.030 (3)	0.029 (2)	0.006 (2)	0.013 (2)	0.019 (2)
C19	0.038 (3)	0.037 (3)	0.041 (3)	0.013 (2)	0.022 (2)	0.013 (2)
C20	0.038 (3)	0.029 (3)	0.039 (3)	0.013 (2)	0.019 (2)	0.021 (2)
N12	0.031 (2)	0.027 (2)	0.032 (2)	0.0085 (16)	0.0155 (17)	0.0119 (17)
O2	0.044 (2)	0.079 (3)	0.046 (2)	0.0153 (18)	0.0187 (17)	0.028 (2)

### Geometric parameters (Å, º)

**Table:**

Cu1—O1	1.929 (3)	N11—C11	1.338 (5)
Cu1—O3	1.930 (3)	N11—C15	1.345 (5)
Cu1—N12	1.984 (3)	C11—C12	1.371 (5)
Cu1—N11	2.006 (3)	C11—H11	0.9500
Cu1—Cl1	2.5944 (12)	C12—C13	1.387 (5)
O1—C1	1.270 (4)	C12—H12	0.9500
C1—C2	1.369 (5)	C13—C14	1.372 (5)
C1—C1^i^	1.537 (7)	C13—H13	0.9500
C2—C3	1.424 (5)	C14—C15	1.383 (5)
C2—H2	0.9500	C14—H14	0.9500
O3—C3	1.275 (4)	C15—C16	1.481 (5)
C3—C4	1.485 (5)	C16—N12	1.371 (5)
C4—C5	1.390 (5)	C16—C17	1.384 (5)
C4—C9	1.412 (5)	C17—C18	1.396 (5)
C5—C6	1.374 (6)	C17—H17	0.9500
C5—H5	0.9500	C18—C19	1.393 (5)
C6—C7	1.378 (6)	C18—H18	0.9500
C6—H6	0.9500	C19—C20	1.375 (5)
C7—C8	1.385 (6)	C19—H19	0.9500
C7—H7	0.9500	C20—N12	1.352 (5)
C8—C9	1.378 (5)	C20—H20	0.9500
C8—H8	0.9500	O2—H2A	0.82 (5)
C9—H9	0.9500	O2—H2B	0.98 (4)
			
O1—Cu1—O3	93.44 (11)	C4—C9—H9	119.4
O1—Cu1—N12	89.27 (12)	C11—N11—C15	118.8 (3)
O3—Cu1—N12	167.52 (12)	C11—N11—Cu1	125.9 (3)
O1—Cu1—N11	163.37 (13)	C15—N11—Cu1	115.3 (3)
O3—Cu1—N11	93.59 (12)	N11—C11—C12	122.1 (4)
N12—Cu1—N11	80.78 (13)	N11—C11—H11	118.9
O1—Cu1—Cl1	95.59 (9)	C12—C11—H11	118.9
O3—Cu1—Cl1	94.10 (9)	C11—C12—C13	119.3 (4)
N12—Cu1—Cl1	97.76 (10)	C11—C12—H12	120.4
N11—Cu1—Cl1	98.92 (9)	C13—C12—H12	120.4
C1—O1—Cu1	122.0 (2)	C14—C13—C12	118.9 (4)
O1—C1—C2	126.3 (4)	C14—C13—H13	120.6
O1—C1—C1^i^	115.8 (4)	C12—C13—H13	120.6
C2—C1—C1^i^	117.8 (5)	C13—C14—C15	119.1 (4)
C1—C2—C3	125.1 (4)	C13—C14—H14	120.4
C1—C2—H2	117.4	C15—C14—H14	120.4
C3—C2—H2	117.4	N11—C15—C14	121.9 (4)
C3—O3—Cu1	123.6 (3)	N11—C15—C16	114.7 (3)
O3—C3—C2	123.4 (4)	C14—C15—C16	123.5 (4)
O3—C3—C4	116.9 (4)	N12—C16—C17	121.7 (4)
C2—C3—C4	119.7 (4)	N12—C16—C15	113.3 (3)
C5—C4—C9	117.5 (4)	C17—C16—C15	125.0 (3)
C5—C4—C3	119.9 (4)	C16—C17—C18	119.7 (4)
C9—C4—C3	122.6 (4)	C16—C17—H17	120.2
C6—C5—C4	121.2 (4)	C18—C17—H17	120.2
C6—C5—H5	119.4	C19—C18—C17	118.3 (4)
C4—C5—H5	119.4	C19—C18—H18	120.8
C5—C6—C7	120.5 (4)	C17—C18—H18	120.8
C5—C6—H6	119.7	C20—C19—C18	119.4 (4)
C7—C6—H6	119.7	C20—C19—H19	120.3
C6—C7—C8	119.9 (4)	C18—C19—H19	120.3
C6—C7—H7	120.0	N12—C20—C19	122.9 (4)
C8—C7—H7	120.0	N12—C20—H20	118.5
C9—C8—C7	119.7 (4)	C19—C20—H20	118.5
C9—C8—H8	120.2	C20—N12—C16	118.0 (4)
C7—C8—H8	120.2	C20—N12—Cu1	126.2 (3)
C8—C9—C4	121.2 (4)	C16—N12—Cu1	115.9 (3)
C8—C9—H9	119.4	H2A—O2—H2B	96 (4)
			
Cu1—O1—C1—C2	−16.0 (6)	C11—C12—C13—C14	−0.4 (6)
Cu1—O1—C1—C1^i^	165.1 (3)	C12—C13—C14—C15	0.1 (6)
O1—C1—C2—C3	−4.0 (7)	C11—N11—C15—C14	1.3 (6)
C1^i^—C1—C2—C3	174.9 (4)	Cu1—N11—C15—C14	−179.5 (3)
Cu1—O3—C3—C2	12.9 (5)	C11—N11—C15—C16	179.4 (3)
Cu1—O3—C3—C4	−168.5 (2)	Cu1—N11—C15—C16	−1.4 (4)
C1—C2—C3—O3	5.8 (6)	C13—C14—C15—N11	−0.6 (6)
C1—C2—C3—C4	−172.7 (4)	C13—C14—C15—C16	−178.5 (4)
O3—C3—C4—C5	15.4 (6)	N11—C15—C16—N12	1.4 (5)
C2—C3—C4—C5	−166.0 (4)	C14—C15—C16—N12	179.5 (3)
O3—C3—C4—C9	−165.8 (4)	N11—C15—C16—C17	179.8 (4)
C2—C3—C4—C9	12.9 (6)	C14—C15—C16—C17	−2.1 (6)
C9—C4—C5—C6	−0.7 (6)	N12—C16—C17—C18	−1.8 (6)
C3—C4—C5—C6	178.2 (4)	C15—C16—C17—C18	179.9 (4)
C4—C5—C6—C7	1.2 (7)	C16—C17—C18—C19	1.8 (6)
C5—C6—C7—C8	−0.4 (7)	C17—C18—C19—C20	−1.3 (6)
C6—C7—C8—C9	−0.8 (6)	C18—C19—C20—N12	0.6 (6)
C7—C8—C9—C4	1.3 (6)	C19—C20—N12—C16	−0.4 (6)
C5—C4—C9—C8	−0.6 (6)	C19—C20—N12—Cu1	179.9 (3)
C3—C4—C9—C8	−179.4 (4)	C17—C16—N12—C20	1.0 (6)
C15—N11—C11—C12	−1.7 (6)	C15—C16—N12—C20	179.5 (3)
Cu1—N11—C11—C12	179.3 (3)	C17—C16—N12—Cu1	−179.2 (3)
N11—C11—C12—C13	1.2 (6)	C15—C16—N12—Cu1	−0.7 (4)

Symmetry code: (i) −*x*, −*y*+1, −*z*+2.

### Hydrogen-bond geometry (Å, º)

**Table:**

*D*—H···*A*	*D*—H	H···*A*	*D*···*A*	*D*—H···*A*
O2—H2*A*···Cl1	0.82 (5)	2.41 (5)	3.224 (3)	171 (5)
O2—H2*B*···Cl1^ii^	0.98 (4)	2.33 (5)	3.307 (4)	172 (4)

Symmetry code: (ii) −*x*, −*y*, −*z*+1.
